# Relationship between disease activity status or clinical response and patient-reported outcomes in patients with non-radiographic axial spondyloarthritis: 104-week results from the randomized controlled EMBARK study

**DOI:** 10.1186/s12955-019-1260-4

**Published:** 2020-01-03

**Authors:** Maxime Dougados, Désirée van der Heijde, Wen-Chan Tsai, Diego Saaibi, Lisa Marshall, Heather Jones, Ron Pedersen, Bonnie Vlahos, Miriam Tarallo

**Affiliations:** 1 Paris Descartes University, Department of Rheumatology, Hôpital Cochin, Assistance Publique - Hôpitaux de Paris, INSERM (U1153), Clinical epidemiology and biostatistics, PRES Sorbonne Paris-Cité, Paris, France; 2 Leiden University Medical Center, Leiden, The Netherlands; 3 Kaohsiung Medical University, Kaohsiung City, Taiwan; 4 Medicity S.A.S, Bucaramanga, Colombia; 5 Pfizer, Collegeville, PA USA; 6 Pfizer, Rome, Italy

**Keywords:** Axial spondyloarthritis, Non-radiographic axial spondyloarthritis, Patient-reported outcome measures

## Abstract

**Background:**

We assessed the external validity of composite indices Ankylosing Spondylitis Disease Activity Score (ASDAS), Bath Ankylosing Spondylitis Disease Activity Index (BASDAI), and Assessment in SpondyloArthritis international Society (ASAS) 40 response (ASAS40) by evaluating the correlations between the changes in some patient reported outcomes (PROs) for patients with non-radiographic axial spondyloarthritis (nr-axSpA) and the changes in the scores of the composite indices.

**Methods:**

This was a post-hoc analysis of data from the EMBARK study in patients with nr-axSpA treated with etanercept. PROs were grouped according to ASDAS status (inactive [< 1.3], low [≥ 1.3 to < 2.1], high [≥ 2.1 to ≤3.5], and very high [> 3.5]), patient achievement of > 50% improvement in BASDAI (BASDAI50 responders), and > 40% improvement in ASAS (ASAS40 responders) at 104 weeks. Analyses were conducted on observed cases available at Week 104. Changes in PROs from Baseline to Week 104 were assessed using analysis of covariance with adjustment for baseline with linear contrast.

**Results:**

Higher ASDAS disease activity at 104 weeks was associated with lower long-term improvement from baseline in PROs (e.g., total back pain [visual analog scale, cm (95% confidence interval): − 4.58 (− 4.95, − 4.21), − 3.86 (− 4.28, − 3.43), − 2.15 (− 2.68, − 1.61), and 1.30 (− 0.51, 3.12) for inactive, low, high, and very high ASDAS disease activity, respectively; Multidimensional Fatigue Inventory (MFI) general fatigue: − 4.77 (− 5.70, − 3.84), − 2.96 (− 4.04, − 1.87), − 1.00 (− 2.32, 0.31), and 2.14 (− 2.10, 6.38); all *p* < 0.001)]. BASDAI50 non-responders had less improvement in PROs from Baseline to Week 104 vs. responders (e.g., total back pain: − 1.61 (− 2.05, − 1.18) vs. –4.43 (− 4.69, − 4.18); MFI general fatigue: − 0.01 (− 1.12, 1.09) vs. –4.30 (− 4.98, − 3.62); all *p* < 0.001). ASAS40 non-responders also had less improvement in PROs from Baseline to Week 104 vs. responders (e.g., total back pain: − 1.91 (− 2.30, − 1.52) vs. –4.75 (− 5.05, − 4.46); MFI general fatigue: − 0.63 (− 1.56, 0.30) vs. –4.64 (− 5.37, − 3.91); all *p* < 0.001).

**Conclusion:**

Composite indices are valid for monitoring treatment response and adequately reflect treatment-related changes experienced by patients with nr-axSpA.

**Trial registration:**

ClinicalTrials.gov identifier: NCT01258738. Registered 9 December 2010.

## Background

Radiographic axial spondyloarthritis (axSpA) is known to have a substantial impact on patients’ physical functioning and health-related quality of life (HRQoL) [1]. In contrast, less is known about the impact of non-radiographic axial spondyloarthritis (nr-axSpA). Few studies to date have fully evaluated the long-term relationship between disease activity/clinical response and patient-reported outcomes (PROs) in patients with nr-axSpA. A recent review reported that patients with nr-axSpA have a substantial burden of illness, with a similar level of impairment of physical function, HRQoL, and work capacity as that reported in patients with radiographic disease [2].

The conventional way to assess the clinical outcomes of treatment for axSpA is to use composite indices such as Ankylosing Spondylitis Disease Activity Score (ASDAS) and Bath Ankylosing Spondylitis Disease Activity Index (BASDAI) [3–5]. Although these are useful for monitoring the signs and symptoms of nr-axSpA, both in clinical practice and trials, PROs on the level of pain, fatigue, disability, HRQoL, and work productivity are increasingly important to consider as well. PROs allow further insight into the impact of the disease on patients’ daily lives and the effectiveness of treatments. As such, PRO data should be considered an important measure of the efficacy of treatments used in patients with nr-axSpA. An outstanding question is whether treatment effect assessed by composite indices adequately reflects changes in PROs.

Results from the EMBARK study have demonstrated that patients with early, active, non-steroidal anti-inflammatory drug (NSAID)-resistant nr-axSpA can be treated effectively with the tumor necrosis factor inhibitor etanercept [6], and that the early improvement in clinical outcomes and markers of inflammation is maintained over 104 weeks [7, 8]. Some short-term improvements in PRO measures (up to 24 weeks) have also been noted [9]. This post hoc analysis of Week 104 data from the EMBARK study examined the long-term relationship between composite outcome measures (ASDAS status criteria, 50% improvement in BASDAI [BASDAI50] response criteria, and 40% improvement in ASAS [ASAS40] responder criteria) and PROs to elucidate whether these composite scores reflect PROs.

## Methods

### EMBARK study design and patients

The EMBARK study was a 2-period, phase IIIb trial (ClinicalTrials.gov identifier: NCT01258738), and the full study details have been published previously [6]. Briefly, eligible patients were aged ≥18 to < 50 years, met the Assessment of SpondyloArthritis international Society (ASAS) classification criteria [10] for axSpA but not the modified New York radiographic criteria for ankylosing spondylitis [11], had symptom duration > 3 months but < 5 years, had BASDAI scores ≥4, and had an inadequate response to at least 2 NSAIDs. In the double-blind phase (Period 1), patients were randomized to receive etanercept 50 mg once weekly subcutaneously or placebo for 12 weeks. After completion of Period 1, patients entered an open-label phase (Period 2) during which they received treatment with etanercept 50 mg once weekly up to 104 weeks. Background NSAIDs were allowed throughout, with stable dosage and type required during Period 1.

The EMBARK study was conducted in accordance with International Conference on Harmonisation Guidelines for Good Clinical Practice and the Declaration of Helsinki. Institutional review board approval and written informed consent from all participants were obtained prior to study initiation.

### Post hoc analyses

In order to assess the impact of disease activity status on PROs of pain, fatigue, HRQoL, and work productivity, patients were grouped according to ASDAS status (inactive [< 1.3], low [≥ 1.3 to < 2.1], high [≥ 2.1 to ≤3.5], and very high [> 3.5]) and also according to BASDAI50 and ASAS40 responses, at Week 104, regardless of their treatment group assignment in Period 1.

The PROs assessed in this analysis have been described in full previously [9] and included: patient global assessment, 0–10 cm visual analog scale (VAS); total back pain, 0–10 cm VAS; nocturnal back pain, 0–10 cm VAS; inflammation, 0–10 cm VAS; Multidimensional Fatigue Inventory (MFI) general fatigue, 4–20; EuroQol-5 Dimensions (EQ-5D), 0–100 mm VAS; EQ-5D utility, 0–1; ankylosing spondylitis quality of life (ASQoL), 0–18; 36-item short form health survey (SF-36) physical component summary (PCS), 0–100; SF-36 mental component summary (MCS), 0–100; Work Productivity and Activity Index (WPAI) absenteeism, 0–100%; WPAI presenteeism, 0–100%; WPAI overall work impairment, 0–100%; and WPAI activity impairment, 0–100%.

All analyses were conducted using observed cases available at Week 104. Disease activity level (ASDAS) and clinical response status (BASDAI50 and ASAS40) were defined at Week 104, and only patients with data available at both Baseline and Week 104 were included in the analysis of PROs. Changes from Baseline to Week 104 were compared using an analysis of covariance model adjusted for Baseline values with a linear contrast.

## Results

Patient disposition at Week 104 has been described previously [7]. Briefly, of the 215 randomized patients, 169 completed 104 weeks of treatment. At Baseline, the mean age was 32 years, 40% of patients were women, and the mean duration of disease symptoms was 2.4 years [7].

**Fig. 1:**
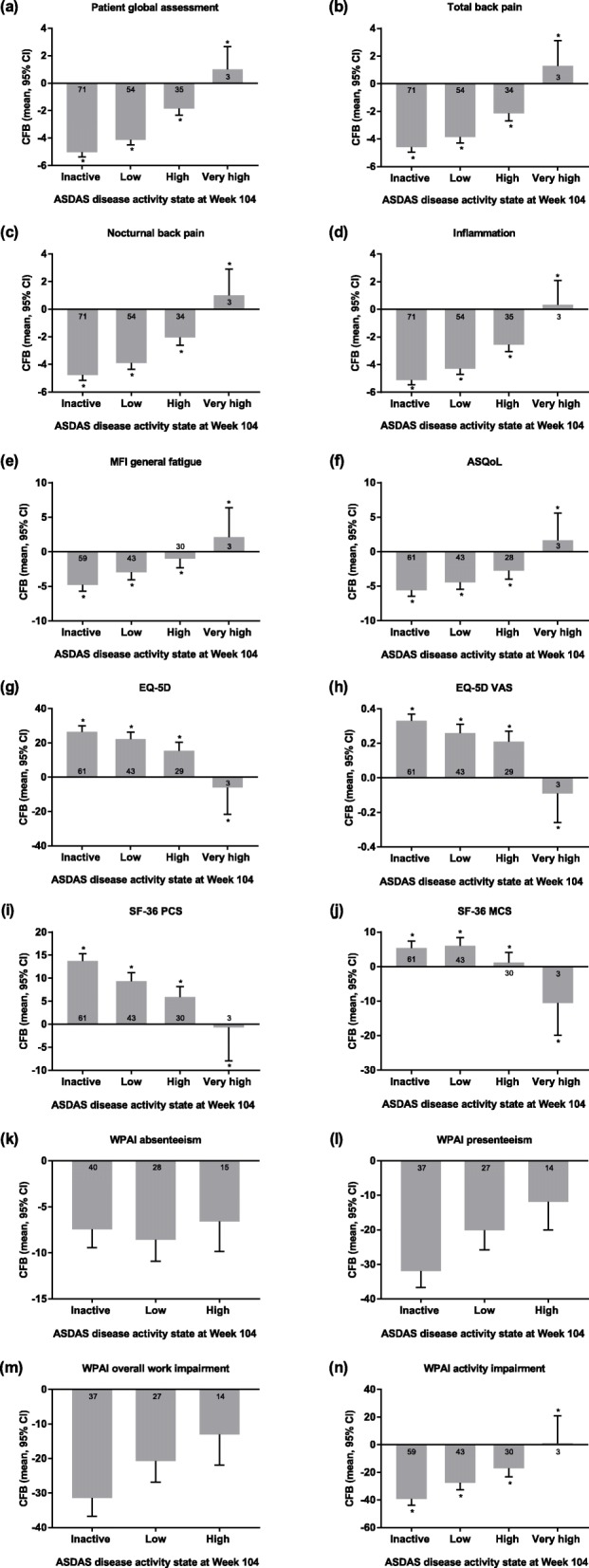
PROs by ASDAS disease activity at Week 104. The response status was defined at Week 104, and only patients with data available at that week were included. Data shown are the adjusted mean change from Baseline (95% CI) except plots for WPAI data, which show adjusted mean change from Baseline in the percentage of patients (95% CI). The number of patients with change from Baseline data are shown in or near the columns. *p < 0.001 for the trend test of adjusted mean change from Baseline. ASDAS, Ankylosing Spondylitis Disease Activity Score; ASQoL, ankylosing spondylitis quality of life; CFB, change from Baseline; CI, confidence interval; EQ-5D, EuroQol-5 Dimensions; MCS, mental component summary; MFI, Multidimensional Fatigue Inventory; NA, not available; PCS, physical component summary; PRO, patient-reported outcome; SF-36, 36-item short form health survey; VAS, visual analog scale; WPAI, Work Productivity and Activity Index

For the ASDAS levels of disease activity at Week 104, the mean changes from Baseline showed that there were improvements in all PROs by Week 104, with significant trends in improved response with lower ASDAS statuses for all of the PROs measured except WPAI absenteeism, presenteeism, and overall work impairment (Fig. 1). There was deterioration in the PROs of the 3 patients who had very high ASDAS at Week 104. Additionally, the data suggest that patients with higher ASDAS at Week 104 had worse patient global assessments, back pain, fatigue, EQ-5D utility, and WPAI presenteeism, overall work activity, and activity impairment scores at Baseline than patients with lower ASDAS at Week 104 had at Baseline; however, this apparent trend could not be ascertained for inflammation, ASQoL, EQ-5D, SF-36, or WPAI absenteeism (see Additional file 1).

**Fig. 2:**
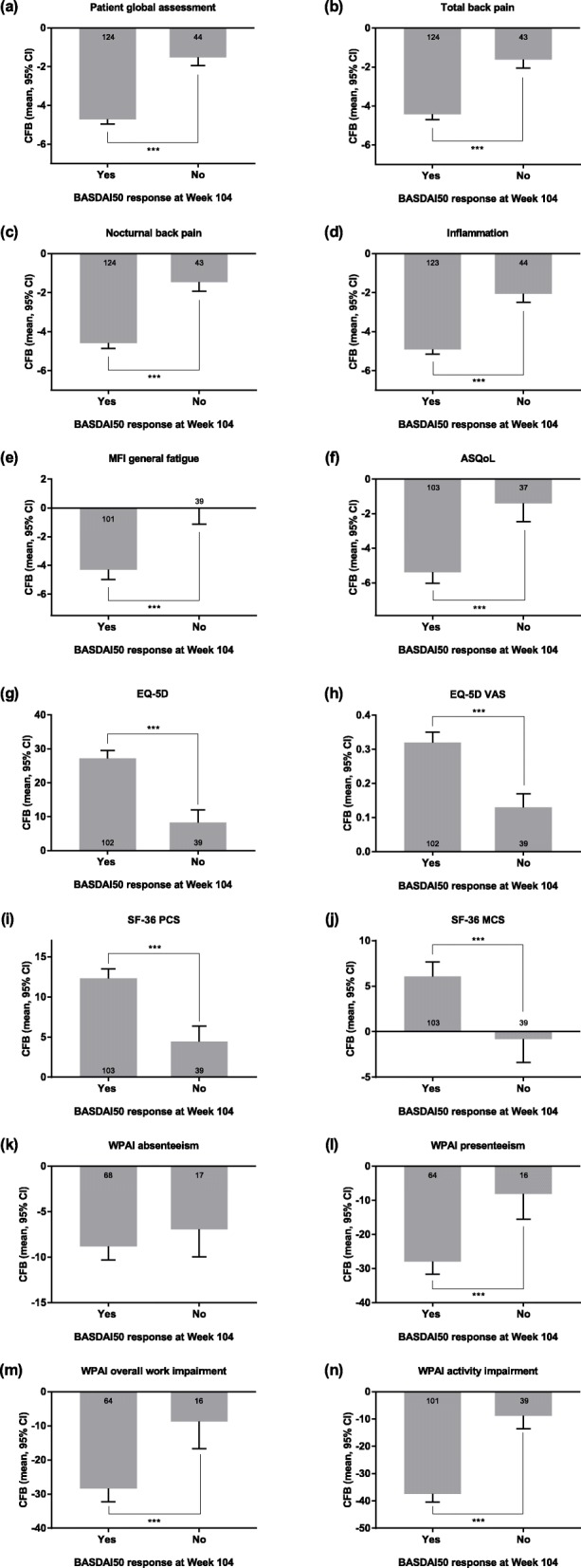
PROs by BASDAI50 response at Week 104. The response status was defined at Week 104, and only patients with data available at that week were included. Data shown are the adjusted mean change from Baseline (95% CI) except plots for WPAI data, which show adjusted mean change from Baseline in the percentage of patients (95% CI). The numbers of patients with available change from Baseline data are shown in or near the columns. Significant difference in change in PRO adjusted mean from Baseline between BASDAI50 responders and non-responders is indicated (***p < 0.001). ASDAS, Ankylosing Spondylitis Disease Activity Score; ASQoL, ankylosing spondylitis quality of life; BASDAI, Bath Ankylosing Spondylitis Disease Activity Index; CFB, change from Baseline; CI, confidence interval; EQ-5D, EuroQol-5 Dimensions; MCS, mental component summary; MFI, Multidimensional Fatigue Inventory; PCS, physical component summary; PRO, patient-reported outcome; SF-36, 36-item short form health survey; VAS, visual analog scale; WPAI, Work Productivity and Activity Index

BASDAI50 responders at Week 104 had significantly greater improvements in mean changes from Baseline compared with non-responders for all of the PROs measured (*p* < 0.001), with the exception of WPAI absenteeism (Fig. 2). BASDAI50 non-responders had mostly minimal improvement in PROs by Week 104, and deterioration was seen for SF-36 MCS. Patients who were BASDAI50 non-responders at Week 104 had worse PROs at Baseline than did patients who were BASDAI50 responders at Week 104, with the exception of patient global assessment, nocturnal back pain, and inflammation (see Additional file 2). However, differences between Baseline PROs for BASDAI50 responders and non-responders were generally small, with the exception of the WPAI items.

**Fig. 3:**
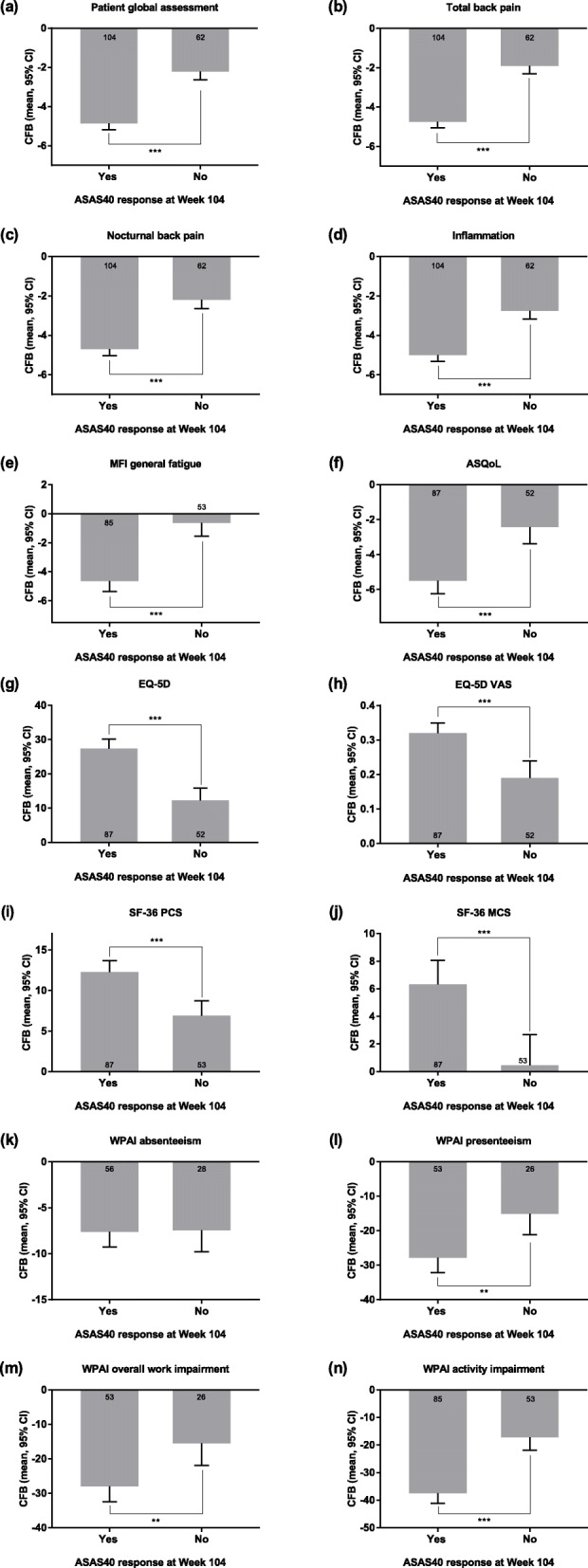
PROs by ASAS40 response at Week 104. The response status was defined at Week 104, and only patients with data available at that week were included. Data shown are the adjusted mean change from Baseline (95% CI) except plots for WPAI data, which show adjusted mean change from Baseline in the percentage of patients (95% CI). The numbers of patients with available change from Baseline data are shown in or near the columns. Significant difference in change in PRO adjusted mean from Baseline between ASAS40 responders and non-responders is indicated (**p < 0.01; ***p < 0.001). ASDAS, Ankylosing Spondylitis Disease Activity Score; ASQoL, ankylosing spondylitis quality of life; BASDAI, Bath Ankylosing Spondylitis Disease Activity Index; CFB, change from Baseline; CI, confidence interval; EQ-5D, EuroQol-5 Dimensions; MCS, mental component summary; MFI, Multidimensional Fatigue Inventory; PCS, physical component summary; PRO, patient-reported outcome; SF-36, 36-item short form health survey; VAS, visual analog scale; WPAI, Work Productivity and Activity Index

ASAS40 responders at Week 104 also had significantly greater improvements in mean changes from Baseline compared with non-responders for all of the PROs measured with the exception of WPAI absenteeism. Significance was at the *p* < 0.01 level for WPAI presenteeism and WPAI overall work impairment, and *p* < 0.001 for all other PROs (Fig. 3). Patients who were ASAS40 responders at Week 104 had worse PROs at Baseline than did patients who were ASAS40 non-responders, with the exception of WPAI absenteeism (see Additional file 3).

## Discussion

The purpose of this post hoc study was to examine whether long-term improvements in composite measures of disease activity and response translate into long-term improvements in patients’ general well-being and work outcomes. The results from the study demonstrated that patients with lower ASDAS states by Week 104 had meaningful improvement in pain, fatigue, physical function, HRQoL, and work productivity. The study also showed that there was less improvement in PROs over time for BASDAI50 and ASAS40 non-responders compared with responders. These data demonstrate a relationship between the composite indices and PROs, and suggest that targeting low ASDAS would also result in optimal improvement in PROs. This validates the use of ASDAS status and/or BASDAI50 and ASAS40 responses as treatment targets with the aim of improving overall HRQoL and reducing impact on the patient’s life.

This relationship reflects observations from other studies in patients with nr-axSpA. For example, in patients with nr-axSpA in the RAPID-axSpA study, an efficacy and safety study of certolizumab pegol in patients with axSpA, improvements in clinical outcomes were mirrored by improvements in PRO measures of sleep, fatigue, and HRQoL by Week 24 and were sustained to Week 204 [12]. A post hoc analysis of data from the ABILITY-1 clinical trial, which assessed the efficacy and safety of adalimumab in patients with nr-axSpA, explored the impact of achieving either an ASAS40 response (40% improvement in ASAS) or various ASDAS states on PRO measures of physical function, HRQoL, and work productivity [13]. In that study, ASAS40 response and ASDAS status were associated with statistically significant and clinically meaningful improvements in the majority of PROs; however, the assessment period was only 12 weeks.

The current study provides evidence that the association of low disease activity status and good clinical response with improved PROs is sustained at 104 weeks in patients with nr-axSpA treated with etanercept. These data further indicate that composite indices adequately reflect PROs in patients with nr-axSpA. Limitations of this analysis include its post hoc nature and the relatively small number of patients in some of the clinical response subgroups. In addition, it should be pointed out that BASDAI, although considered a measure of disease activity, is also a patient-reported instrument. From that perspective, it is not surprising that BASDAI50 response at week 104 was associated with greater improvement in all the PROs assessed, except WPAI absenteeism (Fig. 2).

## Conclusion

In conclusion, Week 104 results from this post hoc study demonstrated that improvements in composite outcomes measures of disease activity and treatment response, which were achieved with etanercept treatment in the EMBARK study, are associated with significant improvements in PROs of pain, fatigue, HRQoL, and work productivity in patients with nr-axSpA. The results from this analysis support the correlation between changes in PRO and changes in composite measures as treatment targets for patients with nr-axSpA, and further contribute to our understanding of how treatment with etanercept for the management of patients with nr-axSpA may improve quality of life and reduce and/or prevent pain and disability.

## Supplementary information

**Additional file 1.** PROs by ASDAS disease activity at Week 104.

**Additional file 2.** PROs by BASDAI50 response at Week 104.

**Additional file 3.** PROs by ASAS40 response at Week 104.

## Data Availability

Upon request, and subject to certain criteria, conditions and exceptions (see https://www.pfizer.com/science/clinical-trials/trial-data-and-results for more information), Pfizer will provide access to individual de-identified participant data from Pfizer-sponsored global interventional clinical studies conducted for medicines, vaccines and medical devices (1) for indications that have been approved in the US and/or EU or (2) in programs that have been terminated (i.e. development for all indications has been discontinued). Pfizer will also consider requests for the protocol, data dictionary, and statistical analysis plan. Data may be requested from Pfizer trials 24 months after study completion. The de-identified participant data will be made available to researchers whose proposals meet the research criteria and other conditions, and for which an exception does not apply, via a secure portal. To gain access, data requestors must enter into a data access agreement with Pfizer.

## Abbreviations

ASAS: Assessment of SpondyloArthritis international Society
ASAS40: 40% improvement in Assessment of SpondyloArthritis international Society score
ASDAS: Ankylosing Spondylitis Disease Activity Score
ASQoL: Ankylosing spondylitis quality of life
axSpA: Axial spondyloarthritis
BASDAI: Bath Ankylosing Spondylitis Disease Activity Index
BASDAI50: 50% improvement in Bath Ankylosing Spondylitis Disease Activity Index score
CFB: Change from Baseline
CI: Confidence interval
EQ-5D: EuroQol-5 Dimensions
HRQoL: Health-related quality of life
MCS: Mental component summary
MFI: Multidimensional Fatigue Inventory
NA: Not available
nr-axSpA,: Non-radiographic axial spondyloarthritis
NSAID: Non-steroidal anti-inflammatory drug
PCS: Physical component summary
PRO: Patient-reported outcome
SF-36: 36-item short form health survey
VAS: Visual analog scale
WPAI: Work productivity and activity index
