# Supplementary material for: Relationship between disease activity status or clinical response and patient-reported outcomes in patients with non-radiographic axial spondyloarthritis: 104-week results from the randomized controlled EMBARK study
Source: Health Qual Life Outcomes. 2020 Jan 3;18:4. doi: 10.1186/s12955-019-1260-4 (PMC6942415; doi:10.1186/s12955-019-1260-4)
Supplement: Supplementary file 1 — Additional file 1. PROs by ASDAS disease activity at Week 104. [file 12955_2019_1260_MOESM1_ESM.docx]

**File S1. PROs by ASDAS disease activity at Week 104**

|  |  | **ASDAS disease activity state at Week 104, mean (95% CI)a** | | | |
| --- | --- | --- | --- | --- | --- |
| **PROs** |  | **Inactive (< 1.3)** | **Low**  **(≥ 1.3 to < 2.1)** | **High (≥ 2.1 to ≤ 3.5)** | **Very high (> 3.5)** |
| Patient global assessment, 0–10 cm VAS | Baseline | 5.48 (4.97, 5.99) | 5.93 (5.32, 6.53) | 6.17 (5.41, 6.93) | 6.87 (4.94, 8.80) |
|  | Wk 104 | 0.74 (0.56, 0.92) | 1.69 (1.34, 2.05) | 4.01 (3.18, 4.85) | 6.80 (4.81, 8.79) |
|  | CFB | –5.02 (–5.36, –4.68)* | –4.12 (–4.50, –3.73)* | –1.85 (–2.33, –1.37)* | 1.00 (–0.66, 2.65)* |
|  | *n* | 71 | 54 | 35 | 3 |
|  | Baseline | 4.91 (4.35, 5.46) | 5.77 (5.09, 6.45) | 5.99 (5.17, 6.80) | 7.63 (4.26, 11.01) |
| Total back pain,  0–10 cm VAS | Wk 104 | 0.83 (0.55, 1.11) | 1.64 (1.27, 2.00) | 3.40 (2.55, 4.26) | 6.83 (3.93, 9.73) |
|  | CFB | –4.58 (–4.95, –4.21)* | –3.86 (–4.28, –3.43)* | –2.15 (–2.68, –1.61)* | 1.30 (–0.51, 3.12)* |
|  | *n* | 71 | 54 | 34 | 3 |
|  | Baseline | 5.15 (4.57, 5.72) | 5.54 (4.81, 6.27) | 5.84 (4.91, 6.77) | 6.27 (0.82, 11.72) |
| Nocturnal back pain,  0–10 cm VAS | Wk 104 | 0.65 (0.36, 0.95) | 1.54 (1.15, 1.93) | 3.45 (2.56, 4.33) | 6.40 (3.78, 9.02) |
|  | CFB | –4.77 (–5.16, –4.38)* | –3.90 (–4.35, –3.46)* | –2.04 (–2.61, –1.48)* | 1.00 (–0.90, 2.91)* |
|  | *n* | 71 | 54 | 34 | 3 |
|  | Baseline | 5.83 (5.28, 6.37) | 6.39 (5.79, 6.99) | 6.13 (5.48, 6.78) | 6.17 (1.02, 11.31) |
| Inflammation,  0–10 cm VAS | Wk 104 | 0.92 (0.69, 1.15) | 1.84 (1.46, 2.21) | 3.56 (2.70, 4.42) | 6.33 (–0.64, 13.30) |
|  | CFB | –5.11 (–5.46, –4.75)* | –4.30 (–4.71, –3.89)* | –2.55 (–3.06, –2.05)* | 0.34 (–1.41, 2.09)* |
|  | *n* | 71 | 54 | 35 | 3 |
|  | Baseline | 14.19 (13.30, 15.07) | 14.40 (13.25, 15.55) | 15.70 (14.53, 16.87) | 19.67 (18.23, 21.10) |
| MFI general fatigue, 4–20 | Wk 104 | 9.64 (8.57, 10.72) | 11.58 (10.40, 12.76) | 14.27 (12.55, 15.98) | 20.00 (─)b |
|  | CFB | –4.77 (–5.70, –3.84)* | –2.96 (–4.04, –1.87)* | –1.00 (–2.32, 0.31)* | 2.14 (–2.10, 6.38)* |
|  | *n* | 59 | 43 | 30 | 3 |
|  | Baseline | 7.33 (6.11, 8.55) | 8.81 (7.23, 10.40) | 8.75 (7.17, 10.33) | 11.33 (5.60, 17.07) |
| ASQoL, 0–18 | Wk 104 | 2.28 (1.41, 3.15) | 4.02 (2.79, 5.26) | 5.64 (3.81, 7.48) | 11.33 (7.54, 15.13) |
|  | CFB | –5.59 (–6.46, –4.71)* | –4.43 (–5.46, –3.40)* | –2.72 (–4.00, –1.44)* | 1.64 (–2.32, 5.60)* |
|  | *n* | 61 | 43 | 28 | 3 |
|  | Baseline | 56.85 (51.72, 61.98) | 60.35 (53.56, 67.14) | 52.72 (45.22, 60.22) | 56.00 (–9.16, 121.16) |
| EQ-5D, 0–100 mm | Wk 104 | 83.66 (80.66, 86.65) | 80.02 (75.70, 84.34) | 71.48 (64.73, 78.24) | 52.33 (–3.56, 108.23) |
|  | CFB | 26.48 (23.04, 29.92)* | 22.20 (18.10, 26.31)* | 15.44 (10.44, 20.45)* | –5.93 (–21.57, 9.71)* |
|  | *n* | 61 | 43 | 29 | 3 |
|  | Baseline | 0.60 (0.53, 0.67) | 0.53 (0.43, 0.63) | 0.52 (0.40, 0.64) | 0.49 (–0.23, 1.21) |
| EQ-5D, 0–1 VAS | Wk 104 | 0.90 (0.86, 0.94) | 0.81 (0.77, 0.86) | 0.76 (0.69, 0.83) | 0.47 (–0.35, 1.28) |
|  | CFB | 0.33 (0.29, 0.37)* | 0.26 (0.22, 0.31)* | 0.21 (0.15, 0.27)* | –0.09 (–0.26, 0.09)* |
|  | *n* | 61 | 43 | 29 | 3 |

|  |  | **ASDAS disease activity state at Week 104, mean (95% CI)a** | | | |
| --- | --- | --- | --- | --- | --- |
| **PROs** |  | **Inactive (<1.3)** | **Low**  **(≥1.3 to <2.1)** | **High (≥2.1 to ≤3.5)** | **Very high (>3.5)** |
|  | Baseline | 37.93 (35.83, 40.04) | 37.93 (35.25, 40.61) | 36.48 (33.12, 39.84) | 30.98 (19.01, 42.95) |
| SF-36 PCS, 0–100 | Wk 104 | 51.40 (49.95, 52.85) | 46.95 (44.66, 49.24) | 42.99 (39.67, 46.32) | 34.28 (19.17, 49.39) |
|  | CFB | 13.74 (12.16, 15.33)* | 9.30 (7.41, 11.18)* | 5.94 (3.67, 8.20)* | –0.69 (–7.95, 6.56)* |
|  | *n* | 61 | 43 | 30 | 3 |
|  | Baseline | 45.97 (43.16, 48.78) | 42.90 (39.62, 46.18) | 42.97 (38.83, 47.11) | 36.11 (21.03, 51.19) |
| SF-36 MCS, 0–100 | Wk 104 | 50.37 (47.72, 53.01) | 49.50 (47.11, 51.89) | 44.85 (40.82, 48.88) | 29.11 (24.89, 33.32) |
|  | CFB | 5.37 (3.32, 7.41)* | 6.01 (3.59, 8.44)* | 1.23 (–1.67, 4.14)* | –10.57 (–19.89, –1.25)* |
|  | *n* | 61 | 43 | 30 | 3 |
|  | Baseline | 8.84 (0.38, 17.30) | 4.85 (–2.51, 12.22) | 16.29 (–1.29, 33.87) | NA |
| WPAI absenteeism, 0–100% | Wk 104 | 1.47 (–1.11, 4.06) | 0.32 (–0.34, 0.99) | 2.01 (–1.18, 5.20) | NA |
|  | CFB | –7.45 (–9.42, –5.48) | –8.57 (–10.92, –6.21) | –6.58 (–9.83, –3.33) | NA |
|  | *n* | 40 | 28 | 15 |  |
|  | Baseline | 35.95 (27.25, 44.64) | 39.63 (29.91, 49.35) | 59.29 (48.55, 70.02) | NA |
| WPAI presenteeism, 0–100% | Wk 104 | 7.84 (4.49, 11.18) | 20.74 (13.98, 27.50) | 35.00 (20.19, 49.81) | NA |
|  | CFB | –31.90 (–36.73, –27.06) | –20.14 (–25.74, –14.55) | –11.85 (–20.00, –3.71) | NA |
|  | *n* | 37 | 27 | 14 |  |
|  | Baseline | 36.57 (27.71, 45.43) | 40.29 (30.48, 50.10) | 61.41 (49.09, 73.72) | NA |
| WPAI overall work impairment, 0–100% | Wk 104 | 8.98 (4.46, 13.49) | 20.94 (14.08, 27.81) | 35.56 (20.25, 50.87) | NA |
|  | CFB | –31.44 (–36.70, –26.19) | –20.74 (–26.81, –14.66) | –12.99 (–21.87, –4.11) | NA |
|  | *n* | 37 | 27 | 14 |  |
|  | Baseline | 46.27 (39.57, 52.97) | 53.49 (45.29, 61.68) | 59.33 (50.79, 67.88) | 73.33 (58.99, 87.68) |
| WPAI activity impairment, 0–100% | Wk 104 | 10.85 (7.15, 14.54) | 24.88 (19.05, 30.71) | 37.33 (27.93, 46.74) | 60.00 (16.97, 103.03) |
|  | CFB | –39.33 (–43.73, –34.92)* | –27.64 (–32.75, –22.53)* | –17.12 (–23.29, –10.95)* | 1.14 (–18.58, 20.86)* |
|  | *n* | 59 | 43 | 30 | 3 |

aData are mean (95% CI) except for WPAI, which are percentage of patients (95% CI).

bMissing 95% CI are due to patients having the same score.

**p* < 0.001 for the test of trend of adjusted mean change from Baseline. The response status was defined at Week 104, and only patients with data available at that week were included. *n* is the number of patients with change from Baseline data. CFB values are the adjusted means.

ASDAS, Ankylosing Spondylitis Disease Activity Score; ASQoL, ankylosing spondylitis quality of life; CFB, change from Baseline; CI, confidence interval; EQ-5D, EuroQol-5 Dimensions; MCS, mental component summary; MFI, Multidimensional Fatigue Inventory; NA, not available; PCS, physical component summary; PRO, patient-reported outcome; SF-36, 36-item short form health survey; VAS, visual analog scale; WPAI, Work Productivity and Activity Index
