# Supplementary material for: Relationship between disease activity status or clinical response and patient-reported outcomes in patients with non-radiographic axial spondyloarthritis: 104-week results from the randomized controlled EMBARK study
Source: Health Qual Life Outcomes. 2020 Jan 3;18:4. doi: 10.1186/s12955-019-1260-4 (PMC6942415; doi:10.1186/s12955-019-1260-4)
Supplement: Supplementary file 2 — Additional file 2. PROs by BASDAI50 response at Week 104. [file 12955_2019_1260_MOESM2_ESM.docx]

**File S2. PROs by BASDAI50 response at Week 104**

| **PROs** |  | **BASDAI50 response at Week 104, mean (95% CI)^a^** | |
| --- | --- | --- | --- |
|  |  | **Yes** | **No** |
| Patient global assessment, 0–10 cm VAS^b^ | Baseline | 5.80 (5.41, 6.18) | 5.72 (5.00, 6.43) |
|  | Wk 104 | 1.07 (0.86, 1.27) | 4.25 (3.62, 4.87) |
|  | CFB | –4.71 (–4.96, –4.46)*** | –1.52 (–1.94, –1.10)*** |
|  | *n* | 124 | 44 |
| Total back pain, 0–10 cm VAS^b^ | Baseline | 5.41 (4.97, 5.84) | 5.65 (4.95, 6.35) |
|  | Wk 104 | 1.03 (0.83, 1.22) | 3.89 (3.20, 4.59) |
|  | CFB | –4.43 (–4.69, –4.18)*** | –1.61 (–2.05, –1.18)*** |
|  | *n* | 124 | 43 |
| Nocturnal back pain, 0–10 cm VAS^b^ | Baseline | 5.57 (5.12, 6.01) | 5.12 (4.31, 5.93) |
|  | Wk 104 | 0.88 (0.67, 1.09) | 3.94 (3.21, 4.67) |
|  | CFB | –4.59 (–4.86, –4.32)*** | –1.46 (–1.92, –1.00)*** |
|  | *n* | 124 | 43 |
| Inflammation, 0–10 cm VAS^b^ | Baseline | 6.18 (5.79, 6.58) | 5.68 (5.05, 6.30) |
|  | Wk 104 | 1.19 (0.98, 1.40) | 3.90 (3.20, 4.59) |
|  | CFB | –4.90 (–5.15, –4.64)*** | –2.06 (–2.49, –1.63)*** |
|  | *n* | 123 | 44 |
| MFI general fatigue, 4–20^b^ | Baseline | 14.15 (13.49, 14.81) | 15.97 (14.80, 17.14) |
|  | Wk 104 | 10.09 (9.30, 10.88) | 15.38 (14.16, 16.61) |
|  | CFB | –4.30 (–4.98, –3.62)*** | –0.01 (–1.12, 1.09)*** |
|  | *n* | 101 | 39 |
| ASQoL, 0–18^b^ | Baseline | 7.68 (6.75, 8.61) | 9.35 (7.89, 10.81) |
|  | Wk 104 | 2.59 (1.94, 3.24) | 7.16 (5.61, 8.71) |
|  | CFB | –5.39 (–6.03, –4.75)*** | –1.39 (–2.46, –0.32)*** |
|  | *n* | 103 | 37 |
|  |  |  |  |
| EQ-5D, 0–100 mm^c^ | Baseline | 57.17 (53.10, 61.23) | 57.05 (50.56, 63.54) |
|  | Wk 104 | 84.37 (82.20, 86.54) | 65.33 (60.32, 70.34) |
|  | CFB | 27.17 (24.87, 29.48)*** | 8.26 (4.54, 11.97)*** |
|  | *n* | 102 | 39 |
| EQ-5D, 0–1 VAS^c^ | Baseline | 0.58 (0.53, 0.64) | 0.51 (0.41, 0.61) |
|  | Wk 104 | 0.89 (0.86, 0.92) | 0.68 (0.62, 0.74) |
|  | CFB | 0.32 (0.29, 0.35)*** | 0.13 (0.08, 0.17)*** |
|  | *n* | 102 | 39 |
| SF-36 PCS, 0–100^c^ | Baseline | 37.97 (36.30, 39.64) | 36.64 (33.90, 39.38) |
|  | Wk 104 | 50.05 (48.80, 51.31) | 41.64 (38.90, 44.38) |
|  | CFB | 12.30 (11.10, 13.50)*** | 4.43 (2.49, 6.37)*** |
|  | *n* | 103 | 39 |
| SF-36 MCS, 0–100^c^ | Baseline | 44.76 (42.61, 46.91) | 42.56 (39.22, 45.90) |
|  | Wk 104 | 50.47 (48.66, 52.29) | 42.65 (39.31, 45.99) |
|  | CFB | 6.08 (4.49, 7.68)*** | –0.79 (–3.38, 1.80)*** |
|  | *n* | 103 | 39 |
| WPAI absenteeism, 0–100%^b^ | Baseline | 8.29 (2.19, 14.38) | 14.73 (–0.72, 30.19) |
|  | Wk 104 | 0.87 (–0.64, 2.37) | 2.31 (–0.62, 5.23) |
|  | CFB | –8.80 (–10.29, –7.32) | –6.94 (–9.94, –3.95) |
|  | *n* | 68 | 17 |
| WPAI presenteeism, 0–100%^b^ | Baseline | 38.75 (32.48, 45.02) | 53.13 (39.41, 66.84) |
|  | Wk 104 | 12.81 (9.41, 16.22) | 36.88 (23.58, 50.17) |
|  | CFB | –27.97 (–31.62, –24.32)*** | –8.10 (–15.55, –0.66)*** |
|  | *n* | 64 | 16 |
| WPAI overall work impairment, 0–100%^b^ | Baseline | 39.63 (33.16, 46.10) | 55.03 (40.06, 69.99) |
|  | Wk 104 | 13.47 (9.71, 17.23) | 37.71 (23.98, 51.44) |
|  | CFB | –28.31 (–32.22, –24.41)*** | –8.68 (–16.66, –0.71)*** |
|  | *n* | 64 | 16 |
| WPAI activity impairment, 0–100%^b^ | Baseline | 49.70 (44.59, 54.82) | 58.21 (50.66, 65.75) |
|  | Wk 104 | 13.86 (11.13, 16.60) | 45.38 (37.66, 53.11) |
|  | CFB | –37.43 (–40.40, –34.47)*** | –8.76 (–13.55, –3.97)*** |
|  | *n* | 101 | 39 |

^a^Data are mean (95% CI) except for WPAI, which are percentage of patients (95% CI).

^b^Higher scores represent worse health or greater impairment.

^c^Lower scores represent worse health or greater impairment.

****p* < 0.001, for the comparison of adjusted mean change from Baseline between BASDAI50 responders and non-responders. Response status was defined at Week 104, and only patients with data available at that week were included. *n* is the number of patients with change from Baseline data. CFB values are adjusted means.

ASDAS, Ankylosing Spondylitis Disease Activity Score; ASQoL, ankylosing spondylitis quality of life; BASDAI, Bath Ankylosing Spondylitis Disease Activity Index; CFB, change from Baseline; CI, confidence interval; EQ-5D, EuroQol-5 Dimensions; MCS, mental component summary; MFI, Multidimensional Fatigue Inventory; PCS, physical component summary; PRO, patient-reported outcome; SF-36, 36-item short form health survey; VAS, visual analog scale; WPAI, Work Productivity and Activity Index.
