# Supplementary material for: Relationship between disease activity status or clinical response and patient-reported outcomes in patients with non-radiographic axial spondyloarthritis: 104-week results from the randomized controlled EMBARK study
Source: Health Qual Life Outcomes. 2020 Jan 3;18:4. doi: 10.1186/s12955-019-1260-4 (PMC6942415; doi:10.1186/s12955-019-1260-4)
Supplement: Supplementary file 3 — Additional file 3. PROs by ASAS40 response at Week 104. [file 12955_2019_1260_MOESM3_ESM.docx]

**File S3. PROs by ASAS40 response at Week 104**

| **PROs** |  | **ASAS40 response at Week 104, mean (95% CI)^a^** | |
| --- | --- | --- | --- |
|  |  | **Yes** | **No** |
| Patient global assessment, 0–10 cm VAS^b^ | Baseline | 6.39 (6.04, 6.73) | 4.66 (4.05, 5.28) |
|  | Wk 104 | 1.12 (0.91, 1.33) | 3.12 (2.49, 3.76) |
|  | CFB | –4.86 (–5.18, –4.55)*** | –2.22 (–2.63, –1.80)*** |
|  | *n* | 104 | 62 |
| Total back pain, 0–10 cm VAS^b^ | Baseline | 6.27 (5.89, 6.65) | 4.08 (3.44, 4.71) |
|  | Wk 104 | 1.06 (0.87, 1.26) | 2.93 (2.29, 3.57) |
|  | CFB | –4.75 (–5.05, –4.46)*** | –1.91 (–2.30, –1.52)*** |
|  | *n* | 104 | 62 |
| Nocturnal back pain, 0–10 cm VAS^b^ | Baseline | 6.14 (5.71, 6.57) | 4.23 (3.57, 4.90) |
|  | Wk 104 | 0.94 (0.74, 1.14) | 2.90 (2.22, 3.58) |
|  | CFB | –4.69 (–5.03, –4.34)*** | –2.19 (–2.64, –1.74)*** |
|  | *n* | 104 | 62 |
| Inflammation, 0–10 cm VAS^b^ | Baseline | 6.67 (6.30, 7.05) | 5.04 (4.48, 5.59) |
|  | Wk 104 | 1.30 (1.04, 1.57) | 2.92 (2.33, 3.52) |
|  | CFB | –5.00 (–5.32, –4.67)*** | –2.75 (–3.17, –2.32)*** |
|  | *n* | 104 | 62 |
| MFI general fatigue, 4–20^b^ | Baseline | 15.00 (14.32, 15.68) | 14.04 (12.96, 15.12) |
|  | Wk 104 | 10.28 (9.39, 11.17) | 13.55 (12.28, 14.81) |
|  | CFB | –4.64 (–5.37, –3.91)*** | –0.63 (–1.56, 0.30)*** |
|  | *n* | 85 | 53 |
| ASQoL, 0–18^b^ | Baseline | 8.89 (7.87, 9.90) | 6.90 (5.67, 8.14) |
|  | Wk 104 | 3.02 (2.19, 3.86) | 5.08 (3.83, 6.32) |
|  | CFB | –5.51 (–6.24, –4.78)*** | –2.43 (–3.38, –1.49)*** |
|  | *n* | 87 | 52 |
| EQ-5D, 0–100 mm^c^ |  |  |  |
|  | Baseline | 53.08 (48.80, 57.36) | 64.83 (59.64, 70.02) |
|  | Wk 104 | 83.64 (81.34, 85.95) | 72.02 (67.01, 77.02) |
|  | CFB | 27.41 (24.68, 30.14)*** | 12.29 (8.72, 15.85)*** |
|  | *n* | 87 | 52 |
| EQ-5D, 0–1 VAS^c^ | Baseline | 0.52 (0.45, 0.59) | 0.63 (0.56, 0.71) |
|  | Wk 104 | 0.87 (0.84, 0.90) | 0.78 (0.72, 0.83) |
|  | CFB | 0.32 (0.28, 0.35)*** | 0.19 (0.15, 0.24)*** |
|  | *n* | 87 | 52 |
| SF-36 PCS, 0–100^c^ | Baseline | 35.86 (34.11, 37.62) | 40.51 (38.22, 42.80) |
|  | Wk 104 | 48.99 (47.47, 50.50) | 46.03 (43.60, 48.46) |
|  | CFB | 12.26 (10.86, 13.67)*** | 6.90 (5.08, 8.72)*** |
|  | *n* | 87 | 53 |
| SF-36 MCS, 0–100^c^ | Baseline | 43.61 (41.21, 46.01) | 45.36 (42.56, 48.17) |
|  | Wk 104 | 50.22 (48.05, 52.40) | 45.28 (42.72, 47.84) |
|  | CFB | 6.32 (4.58, 8.05)*** | 0.45 (–1.77, 2.68)*** |
|  | *n* | 87 | 53 |
| WPAI absenteeism, 0–100%^b^ | Baseline | 7.54 (1.34, 13.74) | 11.13 (–0.14, 22.40) |
|  | Wk 104 | 1.07 (–0.77, 2.90) | 1.37 (–0.36, 3.11) |
|  | CFB | –7.63 (–9.28, –5.97) | –7.45 (–9.79, –5.11) |
|  | *n* | 56 | 28 |
| WPAI presenteeism, 0–100%^b^ | Baseline | 41.89 (34.80, 48.98) | 39.62 (29.30, 49.93) |
|  | Wk 104 | 13.58 (9.29, 17.88) | 25.38 (16.09, 34.68) |
|  | CFB | –27.89 (–32.15, –23.63)** | –15.09 (–21.17, –9.00)** |
|  | *n* | 53 | 26 |
| WPAI overall work impairment, 0–100%^b^ | Baseline | 42.73 (35.49, 49.97) | 40.61 (29.68, 51.54) |
|  | Wk 104 | 14.36 (9.62, 19.09) | 25.94 (16.40, 35.49) |
|  | CFB | –27.97 (–32.49, –23.46)** | –15.49 (–21.94, –9.04)** |
|  | *n* | 53 | 26 |
| WPAI activity impairment, 0–100%^b^ | Baseline | 56.71 (51.26, 62.15) | 44.72 (37.93, 51.50) |
|  | Wk 104 | 16.94 (13.19, 20.69) | 31.13 (24.05, 38.21) |
|  | CFB | –37.47 (–41.12, –33.82)*** | –17.23 (–21.88, –12.59)*** |
|  | *n* | 85 | 53 |

^a^Data are mean (95% CI) except for WPAI, which are percentage of patients (95% CI).

^b^Higher scores represent worse health or greater impairment.

^c^Lower scores represent worse health or greater impairment.

***p* < 0.01, ****p* < 0.001, for the comparison of adjusted mean change from Baseline between BASDAI50 responders and non-responders. Response status was defined at Week 104, and only patients with data available at that week were included. *n* is the number of patients with change from Baseline data. CFB values are adjusted means.

ASDAS, Ankylosing Spondylitis Disease Activity Score; ASQoL, ankylosing spondylitis quality of life; BASDAI, Bath Ankylosing Spondylitis Disease Activity Index; CFB, change from Baseline; CI, confidence interval; EQ-5D, EuroQol-5 Dimensions; MCS, mental component summary; MFI, Multidimensional Fatigue Inventory; PCS, physical component summary; PRO, patient-reported outcome; SF-36, 36-item short form health survey; VAS, visual analog scale; WPAI, Work Productivity and Activity Index.
